# Development and impact of a structured training module for surgical painting and draping among interns

**DOI:** 10.1016/j.infpip.2025.100439

**Published:** 2025-02-07

**Authors:** Tharun Ganapathy Chitrambalam, Abinayaah Suresh, Nidhi Mariam George, Sharmila Aristotle

**Affiliations:** a SRM Medical College Hospital and Research Centre, Chennai, India; b Department of Otorhinolaryngology, Sri Muthukumaran Institute of Technology, Chennai, India; c Department of General Surgery, SRM Medical College Hospital and Research Centre, Chennai, India; d Chettinad Academy of Research and Education, Chennai, India

**Keywords:** Surgical painting and draping, Interns, Surgical site infections, Infection prevention and control, Healthcare-associated infections

## Abstract

**Background:**

Surgical site infections (SSIs) are a global concern affecting patient recovery, prolonging hospital stay and raising healthcare costs.

**Aim:**

To address this, a structured training module was implemented to enhance the efficacy of surgical painting and draping among the interns, reducing SSI risk.

**Methods:**

A questionnaire was distributed to 194 interns, covering fundamental inquiries on SSIs, aseptic practices and an assessment of the participant's knowledge regarding preoperative skin preparation and draping. Students then attended lectures, demonstrations, hands-on sessions and an operating room workshop dedicated to surgical painting and draping techniques. Proficiency was evaluated through a follow-up questionnaire.

**Results:**

There was a significant increase in the percentage of good score from 27.6% to 86.9%. A comparative analysis of surgical site infection (SSI) rates at our institution was performed before and after integrating a cohort of trained clinicians.

**Conclusion:**

Although a significant reduction in SSI rates was observed, it cannot be definitively attributed solely to the introduction of the trained personnel. Nonetheless, the findings underscore the potential impact of rigorous training in aseptic techniques on reducing SSI incidence.

## Introduction

Surgical site infections (SSIs) are infections that occur within 30 days following a surgery (or within one year if an implant remains in place post-procedure). These infections can affect the incision or deeper tissues at the surgical site and can manifest as superficial or deep incisional infections or infections involving the organs and body spaces [1]. In low- and middle-income countries, 11% of surgical patients develop an infection. However, SSIs are not confined to the poorer nations. In the USA alone, they contribute to patients spending more than 400,000 extra days in hospitals with associated healthcare costs exceeding US$900 million annually.

Interns play a pivotal role in mitigating SSIs at the grassroots level. They form the frontline of healthcare and infection prevention efforts. While senior doctors provide guidance, it is the junior doctors who execute their directives. As senior surgeons perform surgeries, the interns are tasked with meticulously preparing the surgical field. The success of any surgery is undermined if the patient develops an SSI, underscoring the critical importance of training our future doctors rigorously in aseptic practices within the operating theatre. The meticulous process of painting and draping the surgical site prior to surgery is an often overlooked art that has been fundamental to surgical practice for the past century. Effective preoperative skin preparation significantly reduces the incidence of SSIs, contingent upon the type of antiseptic utilized and the method of application. Incorrect execution of this procedure may lead to adverse outcomes, potentially facilitating the transfer of micro-organisms from unclean to sterile sites, thereby negating its intended benefits.

The objective of our study was to assess the impact of a structured training module on enhancing the proficiency of interns in the precise techniques of surgical field painting and draping with a concurrent goal of reducing the SSI rates with the hospital.

## Methods

This study was conducted in SRM Medical College Hospital and Research Centre, a 1590-bed facility, equipped with more than 20 operating theatres, where more than 1500 surgeries were performed annually.

Enrolment in the training module included all newly joined interns. The training spanned three days. Day 1 commenced with a pre-training assessment (Supplementary Appendix 1) to gauge the participants' baseline knowledge of SSI sources, antiseptics and basic painting and draping techniques. The participants were subsequently divided into 10 groups, each comprising 20 students. Each group was assigned an expert instructor to facilitate their learning. The participants were then tasked with independently demonstrating four core skills: donning gloves and apron, painting, draping and disposing of waste using the method they deemed most appropriate. Scores were assigned based on their performance consistency: a score of 3 indicated all three attempts were correct, 2 for two correct attempts, 1 for one correct attempt, and 0 for no correct attempts.

Day 2 consisted of a lecture module aligned with the educational standards specified in the *AST standards of practice*. The lecture comprehensively covered SSIs, their sources, the various antiseptics, disinfectants, sterilization techniques available and emphasized effective aseptic practices essential for the operating theatre. Following this, a 2 h formative programme was conducted employing the DOAP teaching method (Demonstration–Observation–Assistance–Performance). Held in a simulation laboratory, this session aimed to immerse students in a clinical environment. Participants learned essential skills such as donning surgical gloves and aprons as well as preparing and draping surgical sites for common elective procedures. The students were instructed in the proper handwashing protocol before gloving for surgery, including washing with antimicrobial soap and water for 15 s to ensure all areas, such as the hands, fingers, and thumbs, were thoroughly covered. After rinsing and drying with a disposable towel, an antiseptic scrub was applied and scrubbed for 6 min. Emphasis was placed on keeping the hands and forearms elevated above the elbows to avoid contamination. Following the current World Health Organization guidelines, students were instructed to use chlorhexidine gluconate with alcohol, recognized for its broad-spectrum antimicrobial activity and prolonged residual effect. They were trained to apply the antiseptic starting at the incision site and moving outward in concentric circles, ensuring thorough and systematic coverage. Emphasis was placed on allowing the antiseptic to dry completely, as this step is critical for maximizing its antimicrobial efficacy and reducing the risk of SSIs. Proper waste disposal techniques were also demonstrated.

Reflecting on their prior performance, students were encouraged to identify areas for improvement. The module featured a comprehensive, step-by-step simulation-based review focusing on precise painting and draping techniques. Deliberate practice was emphasized, with instructors providing constructive feedback throughout. Additionally, participants had the opportunity to observe procedures in a real operating theatre, further enhancing their understanding and readiness for clinical practice.

On day 3 of the module, students underwent a post-training assessment. Their assessment commenced with a written test to determine their understanding and retention of the training material. Following this, the students underwent a detailed evaluation of their practical skills to assess proficiency and application in real-life scenarios. For this, we applied the Direct Observation of Procedural Skills (DOPS) method. Expert evaluators used a checklist to evaluate the proficiency of participants in the four core skills, again with each performed thrice and scored accordingly. Skills were repeatedly practised with immediate feedback until all learners met the required standard. Following the skills assessment, students completed a feedback survey to self-assess their preparedness using a Likert scale ranging from 1 (least prepared) to 7 (most prepared). This comprehensive evaluation ensured that participants not only mastered essential skills but also gauged their confidence and readiness for clinical application (Box 1).

Box 1 **Brief outline of the training programme**

**Table:** 

Day 1
Pre-training assessment
Demonstration of the four core skills
Day 2
Lecture
DOPS session
Operating room workshop
Day 3
Post-training assessment
Demonstration of the four core skills
Feedback survey

Alt-text: Box 1

## Results

A total of 200 interns participated in the training session with 194 individuals successfully completing all days of training, demonstrating high level of commitment and enthusiasm. Notably, none of these participants had prior training in infection prevention practices, highlighting the novelty and potential impact of the programme. Out of the 194 participants who completed the training, 124 were women, constituting 63.9% of the cohort.

The administered pre- and post-test assessments were meticulously analysed. The assessment comprised 15 questions, each carrying a score of 1. The scores were categorized as follows: good: 10–15 points; fair: 5–9 points; poor: 0–4 points.

**Figure 1:**
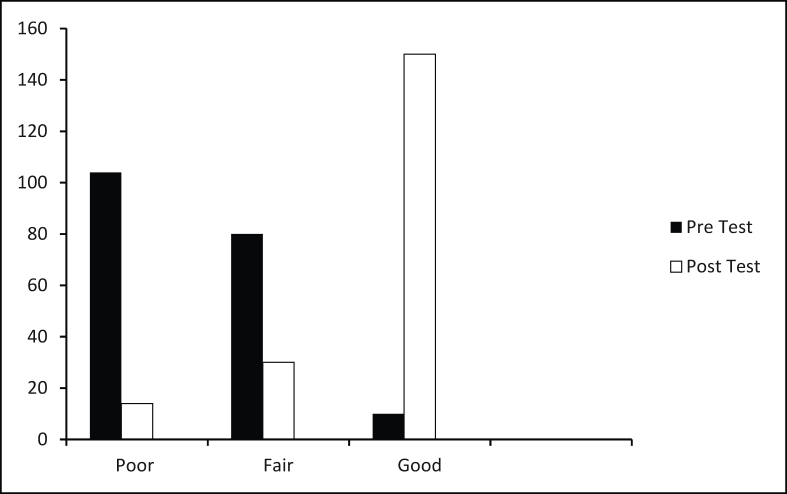
Comparison of scores pre and post test.

A panel of three experts rigorously reviewed the assessment questionnaire for clarity, relevance, comprehensiveness and applicability, ensuring its suitability for evaluating the participants' knowledge acquisition. The pre-test results showed that 104 students scored poorly (0–4), 80 students scored fairly (5–9), and only 10 students scored well (10–15). However, following the intervention, the post-test results demonstrated a remarkable improvement. The number of students scoring poorly fell significantly to 14, while those scoring fairly decreased to 30. Most notably, the number of students scoring well soared to 150. This substantial shift in performance indicates a positive impact of the intervention on the students' academic outcomes (Figure 1).

To determine the statistical significance of this improvement, a χ^2^-test for independence was performed. The test yielded a χ^2^-value of 213.87 with 2 degrees of freedom and *P* < 0.001. This extremely low *P*-value confirms that the observed differences in pre-test and post-test scores are highly statistically significant.

The students were then assessed on the basis of performance of four practical skills: donning gloves and apron, painting, draping, and waste disposal. Each skill was evaluated three times, and the performances were graded on a scale from 0 to 3. A score of 3 indicated that the skill was performed correctly all three times, score of 2 indicated that the skill was performed twice correctly and a score of 1 indicated that the skill was performed once correctly. If the skill was performed all three times incorrectly, it was scored as 0. Post intervention, there was a notable improvement in the mean scores for all skills. To evaluate the statistical significance of these improvements, paired *t*-tests were conducted. The resulting *P*-values were all <0.0001, indicating that the improvements in performance across all skills were highly statistically significant. This demonstrates the effectiveness of the educational intervention in enhancing the students' practical skills.

Feedback was gathered from students using a Likert scale ranging from 1 to 7, where 1 indicated the lowest preparedness and 7 the highest. It was observed that students' confidence levels increased following the training session. They expressed that integrating such a training module would benefit future students, enhancing their preparedness for real hospital settings where mistakes are infrequently tolerated.

The interns who underwent the training subsequently entered their clinical postings. The SSI rates in the hospital during the year these trained individuals were in their clinical postings were then compared with the SSI rates from previous years. While we cannot definitively attribute the reduction in SSI rates solely to our training module, it is plausible that the training had a positive impact on reducing these rates (Table I).

**Table I:** A comparison of the SSI rates in our hospital pre and post intervention

Surgical category	2021	2022	2023 (post-intervention year)
Clean	1.5%	1.4%	0.8%
Clean-contaminated	4.2%	4.0%	2.5%
Contaminated	7.8%	7.5%	5%
Dirty	15%	14.5%	10%

## Discussion

‘Infection is the enemy of progress in medicine; cleanliness is our defence.’ This assertion by Joseph Lister, the pioneer of antiseptic surgery, encapsulates a pivotal advancement in medical history. Prior to the nineteenth century, the link between micro-organisms and wound infections was not recognized [2]. It was Lister's practical application of Louis Pasteur's observations that revolutionized surgical practice, establishing the foundations of antisepsis [3]. The realization emerged that achieving successful surgical outcomes depended not only on the procedure itself but also on maintaining a sterile environment. Just as an artist requires a clean canvas to create a masterpiece, surgeons need a clean field to perform successful operations. This insight highlighted the importance of properly preparing the surgical field through painting and draping techniques. The primary objective of these practices is to reduce the presence of transient microbes and commensal organisms to levels that are insufficient to cause SSIs. The effectiveness of painting and draping is influenced by several factors, including the type of antiseptic used, the necessity of preoperative scrubbing, and whether depilation is required before surgery [4]. Numerous systematic reviews and meta-analyses have been conducted to evaluate these variables. Regardless of the antiseptic chosen, it is crucial that the individual performing the procedure is well-versed in the correct techniques. This is where our study comes into play, addressing this critical aspect of surgical preparation, ensuring that best practices are followed to minimize infection risks.

In most institutions in our country, particularly the teaching institutions, it is uncommon to see the senior-most operating surgeon performing the painting and draping of the surgical field; this task is typically delegated to the junior-most assistant. Furthermore, painting and draping constitute fundamental skills for all doctors, regardless of whether they specialize in surgery or internal medicine. It is crucial to develop these basic skills at the grassroots level to ensure a high standard of practice across all medical disciplines. The implementation of Competency Based Medical Education (CBME) addresses this need by emphasizing student-centred teaching and learning methods. These include the use of skill labs, simulations, guided environments, and small-group teaching and learning, all designed to enhance the proficiency of future medical professionals [5].

In our study, we developed a training module using available resources to effectively prepare the interns before they enter actual operating theatres. Research indicates that many newly graduated doctors feel unprepared to begin working in hospitals and lack confidence in performing basic clinical procedures [6]. We believe that training modules such as ours can bridge this gap. A study by Dutta *et al.* among interns at a medical college in India found that less than half of postgraduate students (43.5%), MBBS students (17.9%), and nursing students (27.3%) adhered to the six steps of handwashing [7]. Similarly, Vaishnav *et al.* demonstrated that prior training in hand hygiene significantly improved participants' knowledge of hand hygiene practices, highlighting the positive impact of training on knowledge acquisition [8]. In 2000, Sherertz *et al.* conducted a one-day course on infection control practices for physicians in training. They observed that the rate of catheter-related infections decreased from 4.51 infections per 1000 patient-days before the course to 2.92 infections per 1000 patient-days 18 months after the course, resulting in estimated cost savings of at least $63,000, potentially exceeding $800,000 [9].

We would like to highlight several common mistakes observed during the training of these interns. Such errors, which are not uncommon, can be mitigated by acknowledging and addressing them. A recurring issue was the tendency of students to touch non-sterile surfaces after gloving and donning aprons. Clear instruction on the distinction between sterile and non-sterile surfaces is crucial. Another frequent mistake was the incorrect technique of painting, where many students moved from peripheral to central areas, often transitioning from unsterile to sterile zones. Additionally, several interns failed to secure their aprons properly, which jeopardized the sterility of the field.

Our study demonstrated a significant increase in participants' knowledge and skills following their attendance at our training module. This approach can be applied to develop other essential skills for upcoming trainees and graduates. As evidenced by our study, even a three-day course can significantly reduce infection rates and overall healthcare costs.

## Conclusion

In conclusion, implementation of a structured course to standardize infection control practices is a cost-effective strategy for reducing hospital-acquired infections. If these results can be reliably reproduced, such a course has the potential to serve as an effective model for training physicians.

## Author contributions

T.G.C.: writing – review and editing, methodology, investigation, formal analysis, data curation, conceptualization. A.S.: writing – review and editing. N.M.G.: conceptualization, writing, methodology, software, resources, data curation. S.A.: formal analysis and data curation. Tharun: writing, writing – review and editing, methodology, investigation, formal analysis, data curation, conceptualization. A.S.: writing – review and editing. N.M. George: conceptualization, writing, methodology, software, resources, data curation. sharmila: formal analysis and data curation.

## Ethics

The Institutional Ethics Committee has approved the study (Reference number: ECR/8926/INST/TN/2013/RR-19).

## Funding sources

None.

## Conflict of interest statement

None declared.
