# Supplementary material for: Development and impact of a structured training module for surgical painting and draping among interns
Source: Infect Prev Pract. 2025 Feb 7;7(2):100439. doi: 10.1016/j.infpip.2025.100439 (PMC11880586; doi:10.1016/j.infpip.2025.100439)
Supplement: Multimedia component 1 [file mmc1.pdf]

# PRE-TEST QUESTIONNAIRE

1. Surgical Site Infection is the leading cause of morbidity and mortality in our country. What is the most common source of pathogen in an SSI.

   A. The exogenous microorganisms
   B. The endogenous flora of the patient's skin
   C. Colonisation and translocation of gastrointestinal microbes
   D. Foreign bodies at the surgical site

2. WHO Global Guidelines recommend the use of alcohol based antiseptic solutions containing Chlorhexidine Gluconate for surgical skin preparation. Which of the following areas must be avoided while using this solution?

   A. Eyes
   B. Middle Ear
   C. Neonates
   D. All of the above

3. What is the minimum accepted duration for surgical hand antisepsis as per standard protocol?

   A. 2 to 6 minutes
   B. less than a minute
   C. At least more than 10 minutes
   D. At least more than 15 minutes

4. Surgical hand preparation is crucial in ensuring asepsis. Identify the INCORRECT step in surgical hand preparation.

   A. Scrub each side of the finger, between the fingers and the back and front of the hand.
   B. Scrub the arms keeping the hand higher than the arm at all times.
   C. Rinse hands and arms by passing them through water in one direction only, from elbow to fingertips.
   D. Proceed to the operating theatre holding hands above elbows.

5. Identify the correct sequence of technique in wearing sterile gloves.

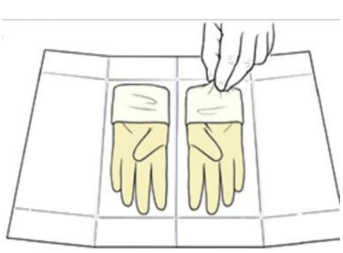

A

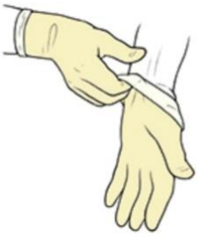

B

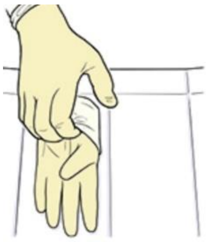

C

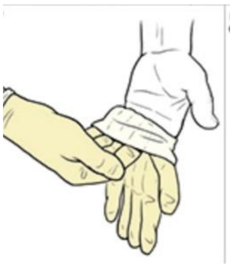

D

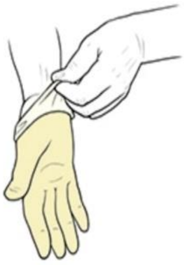

E

A. A, B, C, D, E
B. A, E, C, D, B
C. A, E, B, D, C
D. A, C, D, B, E

6. What is the CORRECT technique of skin preparation when painting a surgical site?

A. Skin prep is begun at the planned incision and carried to the periphery using an ever-widening circular motion
B. Skin prep is begun at the periphery and carried to the site of planned incision using an ever-widening circular motion
C. Skin prep is begun at the axilla/perineum and proceeded in a manner of dirty area to clean area.
D. Skin prep is begun at the planned incision, carried to the periphery and back to the site of the planned incision.

7. The prep solution or paint should not pool under or accumulate near the patient. Why?

   A. In order to prevent inadvertent staining of the patient's body.
   B. To decrease the risk of electrosurgical/laser burns.
   C. This is not a necessary precaution to be adhered to at the time of painting.
   D. This can cause unnecessary interference to the surgeon during surgery.

8. Contaminated areas require special attention while painting. Identify the CORRECT statement regarding surgical painting of contaminated areas.

   A. Areas of high microbial count such as the axilla, perineum, anus and vagina should be prepped first following which the sponge is discarded.
   B. Areas of high microbial count such as the axilla, perineum, anus and vagina should be prepped last following which the same sponge is used for a final paint of the incision site.
   C. Areas of high microbial count such as the axilla, perineum, anus and vagina should not be prepped at the time surgery.
   D. Areas of high microbial count such as the axilla, perineum, anus and vagina should be prepped last following which the sponge is discarded and not reused.

9. What is the minimum number of times of the surgical painting must be done and the duration?

   A. 3 times for 5 minutes
   B. 10 times for 10 minutes
   C. Once for 5 minutes
   D. Once for 10 minutes

10. Drapes create a barrier between the surgical field and possible sources of microbes. Identify the INCORRECT statement in the draping of a surgical site.

    A. Sterile surgical team members must not come into contact with the contaminated undersurface of the drape that has come into contact with a nonsterile surface.
    B. Once a drape has been positioned, it should not be repositioned.
    C. When handling drapes prior to placement on the patient, they should be unfolded.
    D. Surgeon should not reach across an undraped OR table in order to perform a draping procedure.

11. Once the drape has been positioned, a large portion of the drape is noted to be hanging below the edge. Which of the following must NOT be done?

    A. As the part of the drape below the edge is considered to be sterile, it can be readjusted.
    B. As the drape has been positioned into place, it must be not be readjusted.
    C. Only top of furniture, such as the OR table, back table and prep table are considered sterile.
    D. The surgical team member must not come in contact with any contaminated part of the drape.

12. When draping a patient for inguinal hernia surgery, a hole is noticed in the drape. What must be done next?

    A. The draping must be performed in such a manner that the hole is away from the surgical field.
    B. The portion of the drape with the hole must cut off prior to draping.
    C. The entire drape must be discarded.
    D. Draping must be proceeded as before.

13. Once the surgical procedure has been complicated, the gloves and gowns are discarded. Identify the CORRECT statement.

    A. The gloves must always be removed first followed by the gown.
    B. The gown should always be removed first followed by the gloves.
    C. The order of removal of the gown or glove does not affect the sterility.
    D. The gloves must be thrown/sling-shot into the waste container followed by gown removal.

14. Following removal of the surgical gown, where should it be disposed?

    A. The gown must be thrown from a distance into the disposal bag.
    B. The gown must be disposed into the blue coloured impervious disposal bag.
    C. The gown must be disposed into the black coloured impervious disposal bag.
    D. The gown must be disposed into the red/yellow impervious disposal bag.

15. What is to be done following the removal of surgical gowns and gloves?

    A. Immediate medical hand wash has to be performed
    B. Immediate examination of the patient and surgical site
    C. Immediate labelling of the surgical specimen
    D. Immediate documentation of the surgical procedure
